# Graph embedding-based novel protein interaction prediction via higher-order graph convolutional network

**DOI:** 10.1371/journal.pone.0238915

**Published:** 2020-09-24

**Authors:** Ze Xiao, Yue Deng

**Affiliations:** School of Computer Science and Technology, Xidian University, Xi’an, Shaanxi, China; Technische Universitat Dresden, GERMANY

## Abstract

Protein–protein interactions (PPIs) are essential for most biological processes. However, current PPI networks present high levels of noise, sparseness and incompleteness, which limits our ability to understand the cell at the system level from the PPI network. Predicting novel (missing) links in noisy PPI networks is an essential computational method for automatically expanding the human interactome and for identifying biologically legitimate but undetected interactions for experimental determination of PPIs, which is both expensive and time-consuming. Recently, graph convolutional networks (GCN) have shown their effectiveness in modeling graph-structured data, which employ a 1-hop neighborhood aggregation procedure and have emerged as a powerful architecture for node or graph representations. In this paper, we propose a novel node (protein) embedding method by combining GCN and PageRank as the latter can significantly improve the GCN’s aggregation scheme, which has difficulty in extending and exploring topological information of networks across higher-order neighborhoods of each node. Building on this novel node embedding model, we develop a higher-order GCN variational auto-encoder (HO-VGAE) architecture, which can learn a joint node representation of higher-order local and global PPI network topology for novel protein interaction prediction. It is worth noting that our method is based exclusively on network topology, with no protein attributes or extra biological features used. Extensive computational validations on PPI prediction task demonstrate our method without leveraging any additional biological information shows competitive performance—outperforms all existing graph embedding-based link prediction methods in both accuracy and robustness.

## Introduction

Protein-protein interactions (PPIs) are crucial in almost every process in a cell. Understanding PPIs is essential to identify cell physiology states that are normal or diseased. Furthermore, knowledge of PPIs can significantly facilitate uncharacterized protein function prediction and drug design [1, 2]. We usually represent the totality of PPIs in a cell or an organism with a PPI network. These networks are mathematical constructs where every protein is represented as a node, with an edge indicating that two proteins interact. With the development of high-throughput experimental technologies [3–6] (e.g., large-scale PPI screening tools) for the determination of PPIs, an increasing amount of PPI data has become available and provide valuable resources for understanding cell work mechanisms from PPI networks. Despite this, current PPI networks are still incomplete and noisy since the experimental determination of PPIs is limited in how many legitimate interactions they can detect and often have high false positive and negative rates [7]. Moreover, existing experimental maps of PPIs are both expensive and time-consuming. Consequently, these limitations have enabled a series of network-based algorithms [8–12, 15, 17] for predicting missing (unknown) links based on already mapped interactions in noisy and incomplete PPI networks. One class of simple yet efficient approaches for link prediction is called network structured-based algorithms, rooted in social network analysis. Network structure-based algorithms [13–18] such as common neighbors (CN), preferential attachment (PA), Adamic-Adar (AA) and the number of 3-hop paths (*L*_3_), which assign a likelihood score to all candidate links (i.e., non-connected node pairs) and rank these unknown links according to their scores. Due to the simplicity and low computational complexity of network-based algorithms, they have obtained wide practical uses (e.g., Identification of novel protein interactions). In addition, several traditional network embedding algorithms (e.g., multidimensional scaling (MDS) and isometric feature mapping (Isomap)) have been introduced to PPI networks for novel protein interaction prediction. For example, [8] proposed a protein embedding method based on MDS for identifying and assessing new PPIs. Instead of MDS, [10] utilized Isomap to learn latent protein representations by preserving geodesic distances between proteins. The PPI prediction task was transformed to calculate a reliability index for each protein pair. [9] proposed minimum curvilinear embedding (MCE) that converted network topology by leveraging the minimum spanning tree, which outperformed MDS and Isomap in predicting PPIs.

The recently proposed graph embedding [19–22] (sometimes called network representation learning) technologies provide new effective paradigms in graph analysis tasks. Specifically, graph embedding utilizes random walk or matrix factorization to convert network structure into a low-dimensional space in which the topological information of a graph is maximally preserved, often without using node features [23]. In this way, nodes (edges and/or subgraph) of graphs are represented as compacted yet informative vectors in the embedding space and thus can be used as latent features in building traditional machine learning models for a wide range of downstream tasks, such as link prediction and node (or graph) classification. It is worth noting that instead of these advanced graph embedding algorithms, the previous embedding methods, MDS and Isomap, learn node representation by preserving the Euclidean distances of node pairs in the embedding spaces. The main problem with these traditional embedding method is that they need to compute the shortest lengths of node pairs [25]. Although the main application of the recently proposed graph embedding technologies on non-biological networks, such as social networks and recommendation system, numerical experiments indicate that these advanced approaches can also be applied in network biology fields (e.g., drug-disease association (DDA) prediction and PPI prediction) and achieve superior results [24, 25]. The performance of graph embedding and its downstream tasks strongly depend on the type of structure (local and global) property to preserve [25]. Unfortunately, existing graph embedding mainly focuses on capturing local network structural information and fails to exploit a stronger notion of global network topology. Although several embedding approaches (e.g., node2vec [20], LINE [26] and SDNE [27]) are designed to preserve both local and global structure properties, they only consider the first- or second-order similarity of graphs (i.e., 1 or 2-hop neighbors of nodes) for learning node representation. In fact, some factorization matrix-based models (e.g., HOPE [21] and GraRep [22]) aim at preserving the higher-order proximity of networks. However, how to balance higher-order local and global graph structural information to benefit biological informatics, especially novel PPI prediction, is rarely discussed.

Alternatively, various neural network-based methods [26–29], such as LINE, SDNE and VGAE, have also been generalized to the field of graph embedding and show their effectiveness in modeling graph-structured data. A recent important development is graph convolutional networks (GCN) proposed by [28], which have emerged as a powerful architecture for learning node (or graph) representations. Specifically, GCN follows a message passing procedure (i.e., neighborhood aggregation scheme), where each node representation vector is derived by recursively aggregating and transforming the representation vectors of its neighbors. After *L* iterations of the neighborhood aggregation procedure, each node learns a representation vector that captures the network topological information within its *L*-hop neighborhood, where *L* is the number of convolutional layers. These representations can be used as features for various node-related tasks (e.g., link prediction and node classification). Due to the flexibility and effectiveness of GCN, numerous GCN variants have been proposed and garnered particular attention in biological informatics fields [30–32]. For example, several works utilize attention [33, 34], edge features [35] and random walk [36] to improve the basic message passing procedure. [30] adopted a standard GCN decoder and an inner product decoder to discover novel PPIs. However, all of these methods for learning node representation are based on a limited neighborhood aggregation scheme that only considers the immediate (1-hop) neighbors of each node. A larger (higher-order) neighborhood would desirable to provide the GCN model with more topological information, especially for nodes in an incomplete and sparse graph (e.g., PPI network). Furthermore, because GCN are essentially a type of Laplacian smoothing [36], we exploit the higher-order neighborhood information (i.e., increase the sizes/range of the neighborhood) for learning node latent embedding by simply adding the number of convolutional layers (or aggregation/propagation steps) of GCN model, which will cause oversmoothing. It, therefore, loses its focus on the local neighborhood and deteriorates prediction performance [36, 37]. Hence, designing a graph embedding method that can learn a joint node representation of higher-order local and global graph structures would be a promising direction in biological application scenarios.

In this paper, we propose a novel node (protein) embedding method by combining GCN and PageRank as the latter can significantly improve the GCN’s aggregation scheme, which has difficulty in extending and exploring topological information of networks across higher-order neighborhoods of each node. Building on this novel node embedding model, we then develop an adaption of variational graph auto-encoder (VGAE) [29], called HO-VGAE, for novel PPI prediction, which aims to explore only network topology, no protein attributes or extra biological information used in PPI networks. More precisely, we utilize our proposed higher-order GCN to capture highly-order local and global graph structures and learn a lower-dimensional representation (i.e., latent embedding) for each node. This embedding is then used for reconstruction of the PPI network to discover new interactions between protein pairs. A recent study [37] also proposed a message passing algorithm that combines neural network and PageRank, which separates the neural network from the propagation scheme. The predictions are first generated from each node’s features by a neural network (e.g., standard multilayer perceptron (MLP)) and then propagated using an adaption of personalized PageRank. Unlike in [37], our method utilizes PageRank to explore and propagate graph topological information across the higher-order neighborhood of each node in every convolutional layer, not just 1-hop neighborhood aggregation (e.g., the original GCN model [28]). Obviously, the proposed algorithm by [37] requires abundant (additional) node features and is only suitable for node classification tasks.

In addition, to avoid the incompleteness of the PPI network that may result in an unsatisfactory prediction performance, we propose a co-training technique based on the *L*_3_ principle proposed by [17], which indicates that the more 3-hop paths between two proteins in the PPI network, the more likely that a nonobserved (unknown) but legitimate link (interaction) exists. Specifically, we add some nonconnected protein pairs between which there are many 3-hop paths as legitimate links (or present/positive edges) to the training dataset to train our model and improve its prediction performance.

As a consequence, extensive computational validations in PPI networks demonstrate our method without leveraging any additional biological information achieve competitive results in both accuracy and robustness and outperforms all existing graph embedding-based models.

## Methods

In this section, the proposed graph embedding-based method of novel protein interaction prediction is described.

### Definition

PPI data produced by high-throughput experimental technologies come in the form of connections between proteins, which can be naturally modeled as a graph, where proteins are represented as nodes and their interactions are represented as edges. Assume we have an undirected and unweighted graph *G* = (*V*, *E*) with *n* = |*V*| nodes. Denoted by $A=\left[a_{ij}\right]\in R^{n\times n}$ is the adjacency matrix of *G*, where *a*_*ij*_ = 1 denotes a present edge, *a*_*ij*_ = 0 denotes a nonconnected node pair (non-edge). Denoted by *D* = *diag*(*d*_1_, *d*_2_, …, *d*_*n*_) is the degree matrix of *A* where *d*_*i*_ = Σ_*j*_*a*_*ij*_. Node (protein) features are summarized in an *n* × *d* matrix *X*.

### A novel node embedding model with higher-order GCN

Message passing algorithms like GCN [28] are essentially a type of Laplacian smoothing [35], so learning a node representation by recursively aggregating its neighbors’ information could cause oversmoothing if too many layers (or aggregation/propagation steps) are used. This is why the traditional GCN model cannot be trivially expanded to leverage the higher-order neighborhood information of each node.

To effectively capture the larger neighborhood information for each node, we propose a novel mode embedding method by combining GCN and a personalized PageRank algorithm as the latter can significantly improve the GCN’s aggregation scheme, which has difficulty in extending and exploring topological information of networks across higher-order neighborhoods of each node. This higher-order GCN consists of neighborhood aggregation layers that not only consider the immediate neighbors of the nodes but also the higher-order neighborhood. Specifically, our method connects the random walk-based propagation effect (scheme) of personalized PageRank [38] to GCN consecutively in every convolutional layer. We define the root node (i.e., the random walk’s starting node) with a chance of teleporting back (i.e., restart probability *α* ∈ (0, 1]), which ensures the balance of the need to preserve locality and the topological information from the higher-order neighborhood. Note that we assume the diagonal elements of the adjacency matrix *A* set to 1 (i.e., every node is connected to itself) during the following calculations. The nonlinear functions of every convolutional layer can be written as follows:

$$\begin{array}{c} Z^{l+1}=ReLU\left(H_{K}^{l}W^{l}\right), \end{array}$$ (1)

In the *l*-th convolution layer, each power iteration (random walk/propagation) formula is calculated as follows:

$$\begin{array}{c} H_{k+1}^{l}=\left(1-\alpha \right)A_{norm}H_{k}^{l}+\alpha Z^{l}, \end{array}$$ (2)

$$\begin{array}{c} H_{0}^{l}=Z^{l}, \end{array}$$ (3)

where $H_{0}^{0}=X$. *L* is the number of convolution layers. *H*^*l*^ is the matrix of activations in the *l*-th layer, and *W*^*l*^ is the trainable weight matrix in the layer *l*, *ReLU*(⋅) = *max*(0, ⋅) refers to a the activation function of model. $A_{norm}=D^{-\frac{1}{2}}AD^{-\frac{1}{2}}$ is the standard symmetrically normalized adjacency matrix.

In recurrent Eq (2), due to the convergence property of the PageRank algorithm based on random walk, $H_{k+1}^{l}$ is approximately equal to $H_{k}^{l}$ when *k* is large enough. After the *k*-th (*k* → ∞) iteration step, the resulting Eq (2) is

$$\begin{array}{c} H_{\infty }^{l}=\alpha {\left(I_{n}-\left(1-\alpha \right)A_{norm}\right)}^{-1}Z^{l}, \end{array}$$ (4)

where *I*_*n*_ is identity matrix. In the formula above, we also obtain the normalized fully score matrix *S* = *α*(*I*_*n*_ − (1 − *α*)*A*_*norm*_)^−1^, whose element (*S*_*ij*_) specifies the influence score of node *i* on node *j*. Note that due to symmetry *S*_*ij*_ = *S*_*ji*_ (i.e., the influence of *i* on *j* is equal to the influence of *j* on *i*), this inverse always exists and can be used to prove the existence of score matrix *S* [37].

Hence, an adaption of the message passing algorithm of GCNs can be obtained by using the above influence scores, and its nonlinear convolution functions can be written as follows:

$$\begin{array}{c} Z^{l+1}=ReLU\left(SZ^{l}W^{l}\right), \end{array}$$ (5)

$$\begin{array}{c} \text{GCN}\left(\text{X},\text{A}\right)=Z^{L}, \end{array}$$ (6)

**Fig 1:**
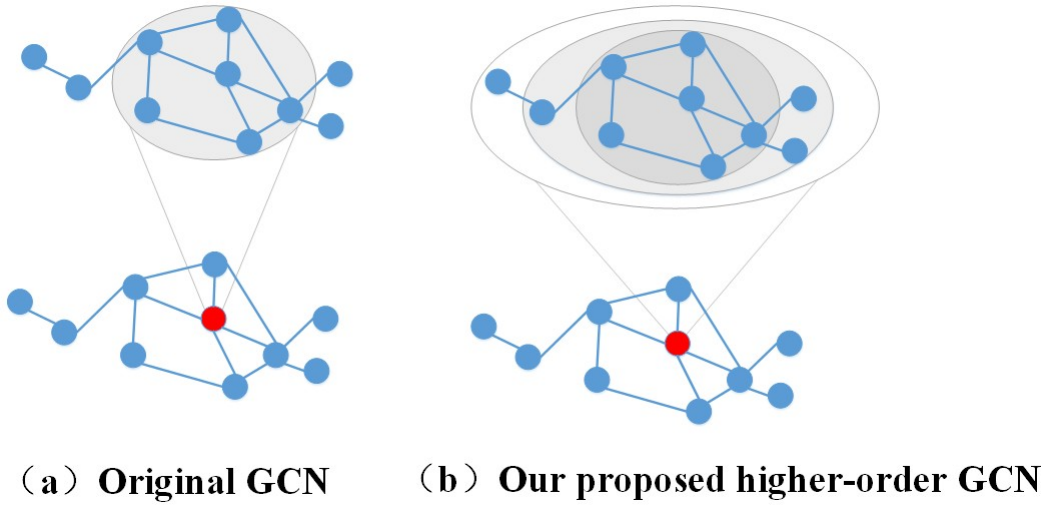
Message passing procedure in original GCNs (a) compared to ours (b). The original GCN follows a 1-hop neighborhood aggregation scheme, which considers only the immediate neighbor information for each node. Our proposed model explores and propagates graph topological information across the highly-order neighborhood of each node in every convolutional layer.

Instead of the standard convolutional formula of the GCN model [28] with a 1-hop neighborhood aggregation scheme, which is calculated via *Z*^*l*+1^ = *ReLU*(*A*_*norm*_ *Z*^*l*^ *W*^*l*^), (7). Our proposed novel embedding model with higher-order GCN is strictly stronger than the original GCN in terms of capturing topological information from a larger neighborhood (as illustrated in Fig 1).

### Higher-order GCN variational auto-encoder for novel protein interaction prediction

Building on our proposed novel node embedding model, we design a higher-order GCN variational auto-encoder (HO-VGAE) architecture developed by [28, 29, 39], which aims to reconstruct a new PPI network to discover novel protein interactions from a noisy and incomplete network.

#### The basic framework

**Fig 2:**
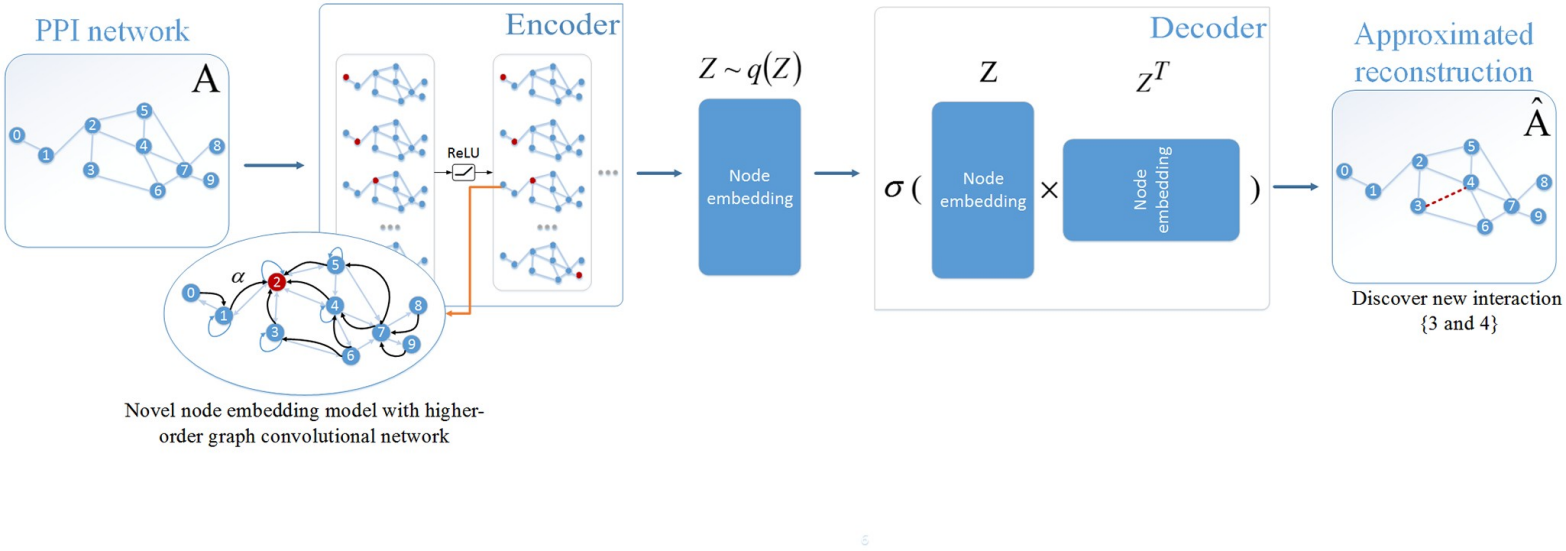
The basic framework of the higher-order GCN variational auto-encoder model to predict new PPIs.

The HO-VGAE architecture consists of multiple nonlinear transforms (mapping functions) on the input structure (i.e., the adjacency matrix *A* and node features matrix *X*), which are summarized in two parts: encoder and decoder. For the encoder part, we utilize our proposed higher-order GCN to generate a Gaussian-distributed latent node embedding $Z\in R^{n\times f}$ from the input structure. This embedding can produce an approximate reconstruction output $\hat{A}$ in the decoder part of the model. The HO-VGAE model focuses on learning low-dimensional and stable node representation preserved graph structural information by minimizing the error between *A* and $\hat{A}$. Our goal is the reconstruction of PPI network, which can be applied to discover new interactions. The basic framework of HO-VGAE is presented in Fig 2. Now, we describe each phase of the HO-VGAE in formal terms.

*Encoder*. The encoder part of HO-VGAE generates low-dimensional latent node embedding. It computes *μ* and *σ*, i.e., two *n* × *f* matrices, by our proposed higher-order GCN:

$$\begin{array}{ccc} \mu ={\text{GCN}}_{\mu }\left(X,A\right) & \text{and} & \text{log}\sigma ={\text{GCN}}_{\sigma }\left(X,A\right), \end{array}$$ (7)

*Embedding*. For each node *i* we can define the distribution of an *f*-dimensional stochastic embedding *Z*_*i*_ by obtained parameters *μ* and *σ* from the encoder:

$$\begin{array}{c} z_{i}|A,X∼N\left(\mu_{i},\mathrm{diag}\left(\sigma_{i}^{2}\right)\right), \end{array}$$ (8)

Hence, given embedding *Z*, we obtain a probability density function for all nodes in the form of an *n* × *f* matrix:

$$\begin{array}{c} q\left(Z|X,A\right)=\prod_{i=1}^{n}q\left(z_{i}|X,A\right), \end{array}$$ (9)

*Decoder*. The decoder part reconstructs the input structure by using the inner product operation between the latent variable representations of two nodes, defined as:

$$\begin{array}{c} p\left(A_{ij}=1|z_{i},z_{j}\right)=\sigma \left(z_{i}^{⊤}z_{j}\right), \end{array}$$ (10)

where $\sigma \left(\cdot \right)=\frac{1}{1+e^{-(\cdot )}}$ refers to logistic function and *σ*(⋅) ∈ (0, 1).

Thus, we can obtain the probability density of a given reconstruction of the PPI network for estimating the likelihood that a nonobserved/unknown link exists, which can be written as:

$$\begin{array}{c} p\left(A|Z\right)=\prod_{i=1}^{n}\prod_{j=1}^{n}p\left(A_{ij}|z_{i},z_{j}\right), \end{array}$$ (11)

*Learning*. The HO-VGAE is trained by optimizing an upper bound:

$$\begin{array}{c} \begin{array}{cc} L & =E_{q(Z|X,A)}\left[\text{log}p\left(A|Z\right)\right]-\text{KL}\left[q\left(Z|X,A\right)∥p\left(Z\right)\right]\\ & =L_{A}+L_{KL} \end{array}, \end{array}$$ (12)

where $L_{KL}$ represents the Kullback-Leibler divergence between the distribution of embedding *q*(Z|X, A) and a Gaussian prior $p\left(Z\right)=\prod_{i}p\left(z_{i}\right)=\prod_{i}N\left(z_{i}|0,I\right)$. The HO-VGAE model performs batch or stochastic gradient descent and utilizes a reparameterization technique for training [39]. Finally, $L_{A}$ and $L_{KL}$ can be expressed directly [39]:

$$\begin{array}{c} L_{A}=\sum_{i,j=1}^{n}\left[A_{ij}\text{log}\left(\sigma \left(z_{i}^{⊤}z_{j}\right)\right)+\left(1-A_{ij}\right)\text{log}\left(1-\sigma \left(z_{i}^{⊤}z_{j}\right)\right)\right)], \end{array}$$ (13)

$$\begin{array}{c} L_{KL}=\frac{1}{2}\sum_{i=1}^{n}\sum_{j=1}^{f}\mu_{ij}^{2}+\sigma_{ij}^{2}-2\text{log}\sigma_{ij}-1, \end{array}$$ (14)

Such a reconstruction process described in the basic framework of the HO-VGAE model is not suitable for directly applying to predict new PPIs because of some characteristics of the PPI network (e.g., incompleteness and sparsity). As we observed, the optimization of the construction of the entire input structure is not intuitive in the case of topological link prediction in an incomplete and sparse PPI network. To solve these issues, we propose a co-training technique and make a few adjustments to loss function L.

#### A co-training technique

The incompleteness of the PPI network problem may make it difficult for our model to accurately identify missing interactions. In the PPI network, we can observe some known interactions (links) between proteins, but simultaneously, many legitimate links are unobserved that are treated as absent (negative) edges. Then, if we train a model by optimizing the object function, which only allows for the contribution of the training model associated with observed interactions (i.e., we directly use the known interactions as the input to a training model), it could lead to an unsatisfactory result. Motivated by the *L*_3_ principle [17], we propose a co-training method to train GCN with more positive samples (known interactions). Specifically, we first use *A*^3^ to find the *t* (i.e., hyper-parameter) most confident unobserved links (edges) *E*′—the *t* most 3-hop paths between two nodes, and then we obtain a new network *G* = (*V*, *E*′ ∪ *E*) with the expanded adjacency matrix *A*′.

Given this expanded adjacency matrix, we refine the learning algorithm of the basic HO-VGAE model as follows by altering the $L_{A}$ (Eq 13) to be optimized.

$$\begin{array}{c} L_{A}=\sum_{i,j=1}^{n}\left[A_{ij}^{\prime }\text{log}\left(\sigma \left(z_{i}^{⊤}z_{j}\right)\right)+\left(1-A_{ij}^{\prime }\right)\text{log}\left(1-\sigma \left(z_{i}^{⊤}z_{j}\right)\right)\right)], \end{array}$$ (15)

Rather than optimizing for the input structure reconstruction outlined in the basic framework of HO-VGAE, we optimize the object function allowing for contribution associated with observed interactions and some unobserved but highly likely legitimate links.

#### Loss adjustments

In practice, the loss function L needs a few adjustments to improve the prediction performance. First, due to the high sparsity of the PPI network, the input structure exhibits an extreme class imbalance in which the number of known links is considerably less than the number of nonconnected links. Then, if we use $L_{A}$ as the loss function to our model, it could result in globally near-zero link reconstruction probabilities. To address this class imbalance issue, we use the density of the network’s adjacency matrix to adjust the $L_{A}$, defined as:

$$\begin{array}{c} {\tilde{L}}_{A}=\sum_{i,j=1}^{n}[\frac{A_{ij}^{\prime }}{d}\text{log}\left(\sigma \left(z_{i}^{⊤}z_{j}\right)\right)+\frac{1-A_{ij}^{\prime }}{1-d}\text{log}\left(1-\sigma \left(z_{i}^{⊤}z_{j}\right)\right))], \end{array}$$ (16)

where $d=\frac{\sum_{ij}A_{ij}^{\prime }}{n^{2}}$. The approach is called balanced cross-entropy, which ensures the positive class and negative class of input structure that contribute equally to the cross-entropy loss formulation.

Second, we add an *L*2-norm regularizer term to prevent overfitting, defined as:

$$\begin{array}{c} L_{reg}=\frac{1}{2}\sum_{l=1}^{L}\left(|\right|W^{l}{\left|\right|}_{F}^{2}), \end{array}$$ (17)

Therefore, the final total reconstruction loss the HO-VGAE is trained for can be written as follows:

$$\begin{array}{c} L_{final}={\tilde{L}}_{A}+L_{KL}+vL_{reg}, \end{array}$$ (18)

#### End-to-end optimization

To improve prediction performance, the HO-VGAE is optimized end-to-end. Our goal is to minimize the objective $L_{final}$ as a function of all trainable parameters (*W*^1^, …, *W*^*L*^). In detail, we first initialize weights using the initialization described in [40]; then, we calculate the partial derivative of $L_{final}$ and obtain updated parameters via back-propagation. Hence, by using the full-batch gradient descent (i.e., propagating loss function gradients through the entire HO-VGAE model, including the encoder part and the decoder part), we can finish the optimization of the HO-VGAE model.

After minimizing the reconstruction loss until the HO-VGAE converges, we can obtain low-dimensional and stable node representation preserved graph structural information. Then, we use the inner product operation ($\hat{A}=\sigma \left({\text{ZZ}}^{T}\right)$) between the latent representations of each node pair to obtain the approximated reconstruction matrix for discovering novel PPIs. In fact, the approximated construction matrix $\hat{A}$ is associated with a confidence score of the interaction and estimates the likelihood that an unobserved or missing link exists. The higher score between two proteins indicates a higher possibility that they interact. The full algorithm is presented in Algorithm 1.

**Algorithm 1** Framework of the HO-VGAE model for novel PPIs prediction.

**Input**: The network *G* = (*V*, *E*) with adjacency matrix *A*

**Output**: The approximated reconstruction matrix $\hat{A}$

1: Replace node (protein) features *X* with the identity matrix in the model;

2: *E*′: the top *t* edges (protein pairs) in *A*^3^;

3: Obtain a new network *G*′ = (*V*, *E*′ *UE*) with adjacency matrix *A*′;

4: Initialize weights(*W*^1^, …, *W*^*L*^) using the initialization described in [40];

5: *Z*^0^ = *X*, $A_{norm}=D^{-\frac{1}{2}}AD^{-\frac{1}{2}}$;

6: Calculate the normalized fully score matrix *S* based on formula *S* = *α*(*I*_*n*_ − (1 − *α*)*A*_*norm*_)^−1^;

7: **repeat**

8:  **for** *l* = 1 to *L* do **do**

9:   *Z*^*l*+ 1^ = *ReLU*(*SZ*^*l*^ *W*^*l*^)

10:  **end** **for**

11:  GCN(X, A) = *Z*^*L*^;

12:  Apply Eq (7) to compute *μ* and *σ*;

13:  $q\left(Z|X,A\right)=\prod_{i=1}^{n}q\left(z_{i}|X,A\right)$ with $z_{i}|A,X∼N\left(\mu_{i},\mathrm{diag}\left(\sigma_{i}^{2}\right)\right)$;

14:  $p\left(A|Z\right)=\prod_{i=1}^{n}\prod_{j=1}^{n}p\left(A_{ij}|z_{i},z_{j}\right)$ with $p\left(A_{ij}=1|z_{i},z_{j}\right)=\sigma \left(z_{i}^{⊤}z_{j}\right)$;

15:  Apply Eqs (15), (13) and (16) to compute ${\tilde{L}}_{A}$, $L_{KL}$ and $L_{reg}$, respectively;

16:  $L_{final}={\tilde{L}}_{A}+L_{KL}+vL_{reg}$;

17:  Use back-propagation to calculate the partial derivative of $L_{final}$ and get updated parameters (*W*^1^, …, *W*^*L*^).

18: **until** converage

19: We obtain stable node (protein) embedding *Z* based on global graph topology;

20: Apply the inner product operation ($\hat{A}=\sigma ({\text{ZZ}}^{T}$) to obtain the approximated reconstruction matrix for estimating the likelihood that a unobserved or missing link exists.

#### Accuracy and efficiency analysis

As we observed when adding the propagation effects of the PageRank algorithm to GCN consecutively during inference (i.e., steps 7-16 in Algorithm 1), PageRank can significantly explore the useful information from higher-order neighborhood for each node and then improve the model’s accuracy. However, because calculating the normalized fully score matrix *S* (step 6 in Algorithm 1), i.e., reconstructing a dense $R^{n\times n}$ matrix, requires $O(n^{2})$ computational complexity and memory, we achieve higher-order GCN by directly using this matrix for training and inference would be computationally inefficient.

To avoid this issue, we can approximate the nonlinear convolution functions (Eq (5)) that utilize a score matrix *S* via PageRank’s power iteration connected to the regular random walk. The approximate computation algorithm for the calculation of GCN(X, A), i.e., steps 8-11 in Algorithm 1, is described in Algorithm 2. Note that this method retains the graph’s sparsity and achieves linear computational complexity.

**Algorithm 2** The approximate computation for the calculation of GCN(X, A).

**Input**: Nodes (proteins) feature matrix *X* and the initialized weights(*W*^1^, …, *W*^*L*^)

**Output**: GCN(X, A)

1: $H_{0}^{0}=Z^{0}=X$, $A_{norm}=D^{-\frac{1}{2}}AD^{-\frac{1}{2}}$

2: **for** *l* = 1 to *L* do **do**

3:  **for** k = 0 to *K* do **do**

4:   *Z*^*l*+ 1^ = *ReLU*(*SZ*^*l*^ *W*^*l*^)

5:  **end** **for**

6:  $Z^{l+1}=H_{0}^{l+1}=ReLU\left(H_{K}^{l}W^{l}\right)$

7: **end** **for**

8: $\text{GCN}\left(X,A\right)=H_{K}^{L}$.

## Results and discussion

In this section, we demonstrate the ability of the HO-VGAE to learn meaningful latent embeddings on novel PPI prediction tasks on several human PPI networks. The experimental results indicate significant improvements in both accuracy and robustness over all existing graph embedding-based link prediction models. We also predicted the novel PPIs by training HO-VGAE using the complete network and then compiled a list of most likely potential PPIs (i.e., top 150 predictions) ranked according to their interaction confidence score for biologists to test (S2 File).

### Datasets

To comprehensively assess the performance of the HO-VGAE, we use six human interactomes curated by [17] for novel PPI prediction. We start from a systematic PPI network, HI-II-14 [4], obtained from a binary pipeline. We then predict new interactions on the HI-III dataset [41] produced by high-throughput experimental PPI screening technologies. We continue with literature-curated PPI networks of direct physical interactions, such as the Lit-BM-13 [6] and BioGRID [42] datasets. We finally consider co-complex proteomics datasets, such as Bioplex [6] and Hein et al [5]. Following [17], we summarized the detailed statistics of the datasets in S1 Table in S3 File.

### Baseline model

We first compare our model with following 8 representative graph embedding techniques.

- Graph Factorization (GF) [43]: It factorizes the Laplacian matrix of the adjacency matrix to obtain graph representation. It only preserves the first-order property of the network.
- GraRep [22]: A recently proposed graph embedding method based on matrix factorization focuses on capturing the high-order proximity of graphs and designing *k*-step transition probability matrices for factorization.
- HOPE [21]: It also takes into account the high-order proximity to preserve graph structures by utilizing heuristic network similarity.
- DeepWalk [19]: It generates network embedding by using a random walk and skip-gram model.
- node2vec [20]: Compared to DeepWalk, node2vec adopts a biased second-order random walk procedure with more flexibility to generate node embedding.
- LINE [26]: It adopts neural network architectures (i.e., MLP) and directly models node embedding vectors by optimizing the loss function, which preserves the 1st-order and/or 2nd-order proximity of graphs.
- SDNE [27]: It utilizes a deep auto-encoder to preserve the second-order proximity by reconstructing the neighborhood structure of each node and incorporate the first-order proximity using the Laplacian Eigenmaps model [44].
- VGAE [29]: It consists of two-layer GCN and a simple inner product decoder to learn meaningful latent embedding based on the variational autoencoder [39].

Besides the above embedding techniques, we also compare our embedding model with 5 state-of-the-art network structure-based link prediction algorithms, including common neighbors (CN), preferential attachment (PA), Adamic Adar (AA), *L*_3_ principle [17] as well as its higher-order extension (CH2-L3) [18].

We can directly apply the HOPE and VGAE model to the PPI prediction task. Following [24], to adapt the other embedding-based baseline algorithms to predict novel PPIs, we first use these graph embedding methods to learn latent node representation. We then concatenate the embeddings of each node pair (i.e., edges and non-edges) as features to build a training model (e.g., Logistic Regression binary classifier) for predicting unknown interaction. Note that another typical operator is the Hadamard product for obtaining the training features of node pairs, we also report the prediction performance using this pipeline in S5 Table and S6 Table in S3 File. The result indicates the concatenation operation outperforms the Hadamard product operator.

### Experimental setup

In the PPI network, all the existing links (interactions) are randomly split into a set of training PPI, validation and test sets. Thus, the model is trained on an incomplete PPI network (i.e., contains 80% of known interactions) where parts of the known links have been removed. We form validation and test set from removed edges (i.e., each includes 10% of known interactions) and a matching number of randomly sampled unlinked protein pairs. The validation set is used for the optimization of hyper-parameters. We use the area under precision recall (AUPR) curve and precision@k (k is the number of 10% of known interactions) to evaluate the link prediction performance. Meanwhile, AUPR is used to choose the best hyper-parameters.

Before the optimization of the HO-VGAE model, we initialize the weights (*W*^1^, …, *W*^*L*^) using the initialization described in [40]. We train for a maximum of 100 iterations using Adam optimizer [45] with a learning rate of 0.01. To prevent overfitting and experimental bias, we use a regular dropout [46] to hidden layer units and apply early stopping with a window size of 2, i.e., we stop training if the validation set loss does not decrease for two consecutive iterations. In addition, notice that our model does not incorporate any additional information about protein attributes, but only network topology, so we can replace node features matrix *X* with the identity matrix in the GCN model. We can also apply the graph embedding techniques (e.g., DeepWalk) to generate node embeddings (features). However, in practice, node features obtained by graph embedding techniques didn’t improve the performance of VGAE and HO-VGAE (S7, S8 Tables in S3 File).

We optimize all hyper-parameters (e.g., *α*, *k*, and *t*) of all embedding models (i.e., HO-VGAE and all embedding-based baseline) for all PPI networks individually using a grid search on the validation set. Specifically, HO-VGAE applies a 3-layer GCN architecture with *d*(1) = 256, *d*(2) = 128 and *d*(3) = 64 hidden units in each convolutional layer and a dropout rate of 0.5 in our experiments. We also report all candidate hyper-parameter values over which the grid search has been performed and default hyper-parameter values in S2 Table in S3 File.

### Accuracy

The results for AUPR are summarized in S3 Table in S3 File. All results were summarized over 10 trials and expressed as mean ± standard deviation. As can be seen, the HO-VGAE significantly outperforms all compared models on all datasets. In particular, we obtain these key conclusions as follows.

Generally, the recently proposed embedding approaches can be applied to novel PPI prediction and are more effective than conventional network structure-based link prediction algorithms (e.g., CN and AA). For example, compared to CN, SDNE achieved 15.9%-36.1% improvements of the AUPR value on all datasets. Our experiments also demonstrate that the VGAE developed by the GCN model and VAE is a promising unsupervised approach for learning powerful latent embeddings for the structure information of networks, which can be used for the prediction of PPI. However, the result indicates that the VGAE with two 2-layer GCN for learning node representations fails to exploit stronger topological information from the higher-order neighborhood of each node, while our proposed HO-VGAE exhibits much superior performance (e.g., HO-VGAE obtains 2.5%-8.1% gains in terms of the AUPR value on the six human PPI networks compared with VGAE) in the novel PPI prediction task, as it overcomes limitations of the VGAE model. In addition, we observe that VGAE with L3 co-training outperforms pure VGAE by large margins. This implies that our proposed co-training method can improve the prediction performance of embedding model. However, it’s also interesting that compared to HO-VGAE without L3 co-training, adding L3 co-training does not significantly improve the performance and does not always improve the performance.

In practice, the known PPI network (matrix) exhibits class (label) imbalance in which the number of known interactions is considerably less than the number of non-interacting pairs. To mimic this situation, we further performed two additional validation tests, in which the negative examples in the test set contained either the negative samples nine times more than the positive ones (i.e., positive-negative ratio of the test set = 1:10) or all unconnected protein pairs in the remaining PPI network. The experiment results under the above settings are summarized in S4 Table in S3 File and Table 1, respectively. In these two experimental scenarios, L3 (or CH2-L3) always show great performance, which indicates they have much better predicting power for identifying novel/unknown PPIs in the extremely sparse networks. It’s interesting that the AUPR values of all graph embedding-based approaches, including our proposed HO-VGAE, dropped greatly when all unknown pairs were treated as negative examples. Despite this, HO-VGAE still obtains a much higher AUPR score and shows superior ability in identifying missing PPIs when compared to other graph embedding-based methods.

**Table 1:** Link prediction performance on six human PPI networks (AUPR). All unknown pairs were treated as negative examples.

Method Name	HI-II-14	HI-III	Lit-BM-13	BioGRID	Bioplex	Hein et al	Mean
CH2-L3	13.9±0.7	**16.3**±**0.3**	**10.5**±**0.5**	**15.6**±**0.3**	**14.0**±**0.3**	**16.5**±**0.3**	14.5
L3	**14.3**±**0.7**	15.6±0.5	10.4±0.8	15.0±0.7	13.8±0.3	16.4±0.7	14.2
CN	3.1±0.2	3.1±0.6	6.0±0.7	5.7±0.3	6.0±0.4	8.4±0.4	5.4
**HO-VGAE+CH2-L3**	4.9±0.3	5.3±0.2	2.4±0.1	5.8±0.1	5.1±0.0	1.6±0.0	4.2
**HO-VGAE+L3**	5.0±0.3	5.3±0.3	2.5±0.2	5.8±0.2	5.0±0.1	1.6±0.0	4.2
**HO-VGAE**	4.7±0.3	5.1±0.2	2.4±0.0	5.7±0.2	4.7±0.1	1.6±0.0	4.1
AA	1.9±0.1	2.2±0.3	2.5±0.2	3.0±0.1	5.0±0.2	6.4±0.1	3.5
**VGAE+L3**	3.6±0.3	4.5±0.1	1.7±0.1	4.6±0.2	3.7±0.1	1.5±0.1	3.2
VGAE	3.4±0.1	4.4±0.1	1.4±0.1	4.5±0.1	3.4±0.1	1.3±0.1	3.1
PA	3.5±0.9	4.0±0.2	1.1±0.1	2.6±0.3	2.6±0.3	1.4±0.1	2.5
GraRep	2.0±0.2	2.3±0.1	1.0±0.1	1.0±0.3	1.6±0.1	0.9±0.1	1.5
HOPE	1.7±0.3	2.0±0.3	0.9±0.0	0.9±0.1	1.3±0.0	0.7±0.0	1.3
LINE	1.2±0.2	1.1±0.2	0.7±0.0	1.1±0.0	1.1±0.0	0.7±0.0	1.0
SDNE	1.1±0.2	1.0±0.1	0.5±0.0	1.3±0.0	1.0±0.0	1.2±0.1	1.0
GF	1.4±0.2	1.7±0.2	0.4±0.0	0.6±0.0	0.9±0.0	0.5±0.0	0.9
DeepWalk	1.0±0.1	1.8±0.1	0.2±0.0	1.2±0.3	0.8±0.0	0.6±0.0	0.9
node2vec	0.8±0.2	1.5±0.1	0.2±0.0	1.0±0.2	0.7±0.0	0.8±0.0	0.8

The results for precison@k (k is the number of 10% of known interactions) are summarized in Table 2. We find that L3 and CH2-L3 outperform other state-of-the-art baseline methods and our proposed HO-VGAE. We also observe that the performance of HO-VGAE is consistently better than other graph embedding-based approaches. Thus, HO-VGAE achieves competitive performance showing that it’s a powerful ability in predicting novel/unknown PPIs.

**Table 2:** Overall link prediction performance comparison on six human PPI networks (Precision@k).

Method Name	HI-II-14	HI-III	Lit-BM-13	BioGRID	Bioplex	Hein et al	Mean
CH2-L3	19.0±0.5	**20.1**±**0.3**	14.6±0.3	9.3±0.2	**14.1**±**0.4**	**26.8**±**0.5**	17.3
L3	**19.2**±**0.3**	19.9±0.2	**15.0**±**0.6**	9.5±0.2	13.5±0.2	26.6±0.3	17.3
**HO-VGAE+CH2-L3**	17.7±0.2	18.8±0.3	10.8±0.1	**10.6**±**0.3**	12.6±0.2	13.0±0.2	13.9
**HO-VGAE+L3**	17.7±0.1	18.5±0.3	10.9±0.1	10.4±0.2	12.6±0.3	13.1±0.5	13.8
**HO-VGAE**	17.6±0.1	18.5±0.4	10.6±0.5	10.4±0.3	12.4±0.3	12.8±0.3	13.7
**VGAE+L3**	15.5±0.2	17.0±0.3	9.1±0.2	9.2±0.4	10.8±0.4	11.7±0.3	12.2
VGAE	15.2±0.4	16.5±0.3	8.9±0.2	9.0±0.2	10.1±0.3	11.2±0.4	11.8
GraRep	12.6±0.3	14.8±0.2	8.0±0.4	9.8±0.3	9.2±0.4	10.8±0.3	10.6
HOPE	12.7±0.6	11.4±0.3	7.8±0.1	7.9±0.3	7.0±0.3	8.1±0.1	9.2
LINE	11.4±0.2	11.0±0.1	6.1±0.3	8.5±0.3	8.6±0.3	9.1±0.3	9.2
SDNE	8.5±0.3	10.2±0.2	4.1±0.2	7.1±0.1	7.4±0.1	7.6±0.2	7.5
GF	7.7±0.2	6.9±0.1	7.7±0.5	5.1±0.3	4.4±0.1	7.3±0.1	6.5
DeepWalk	6.7±0.3	8.9±0.4	3.3±0.0	5.0±0.1	6.0±0.1	5.8±0.2	5.9
AA	4.6±0.3	5.3±0.1	4.8±0.2	2.0±0.0	5.2±0.1	13.1±0.7	5.8
node2vec	4.8±0.1	8.5±0.1	1.5±0.0	3.1±0.2	5.3±0.2	4.3±0.3	4.6
CN	0.0±0.0	5.7±0.1	6.5±0.3	2.1±0.1	4.9±0.0	0.3±0.0	3.2
PA	3.9±0.2	4.5±0.3	2.3±0.0	1.8±0.0	0.7±0.0	3.1±0.1	2.7

### Robustness

**Fig 3:**
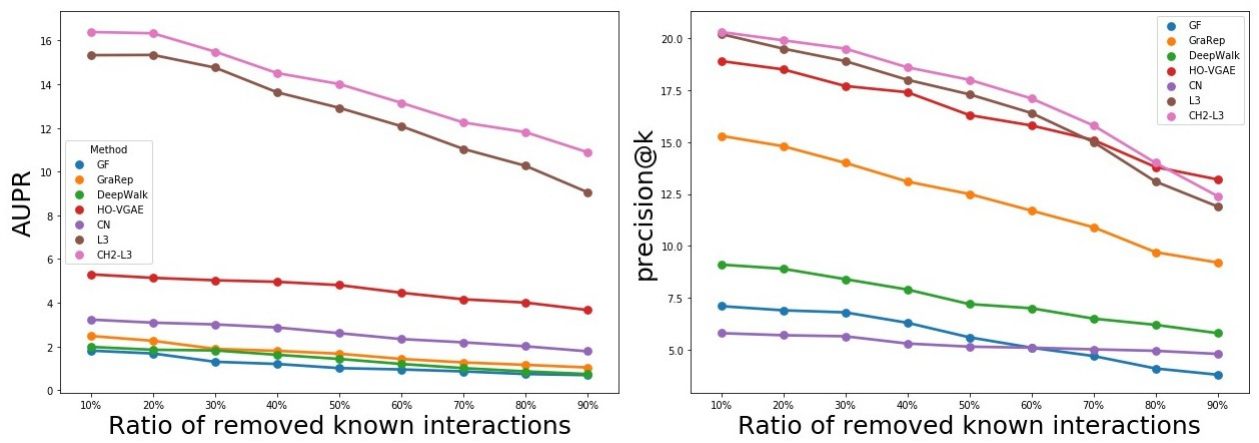
The results of six link prediction methods on the HI-III dataset of different incompleteness.

We illustrate the potential of the HO-VGAE in effectively capturing strong structural information of graphs and predicting PPI in the presence of noise. In particular, to explore the robustness of HO-VGAE, we randomly remove 10%, 20%…, 80%, and 90% known interactions (links/edges) from the PPI network, respectively, and then follow the aforementioned procedure to report the results of different link prediction methods. Note that all prediction approaches didn’t use the co-training technique and all non-interacting pairs were treated as negative examples. The result (AUPR and precision@k) on the HI-III dataset is shown in Fig 3.

We find that the predictive power of the HO-VGAE model persists even when over 60%-70% of the known links are removed. Additionally, our model performs stably better than baseline algorithms (except for L3 and CH2-L3) up to the removal of 90% of links of the PPI network. It also demonstrates that the great power of HO-VGAE in predicting potential (missing) links with incomplete (noisy) networks.

### Effects of parameters

In this section, we investigate the effects of hyper-parameters on the performance of PPI prediction. Specifically, we report the AUPR on three PPI datasets (HI-III, Lit-BM-13 and Hein at al) to evaluate how different values of hyper-parameters *α* and *K* can affect the prediction results. Note that HO-VGAE didn’t use the co-training technique and all non-interacting pairs were treated as negative examples.

**Fig 4:**
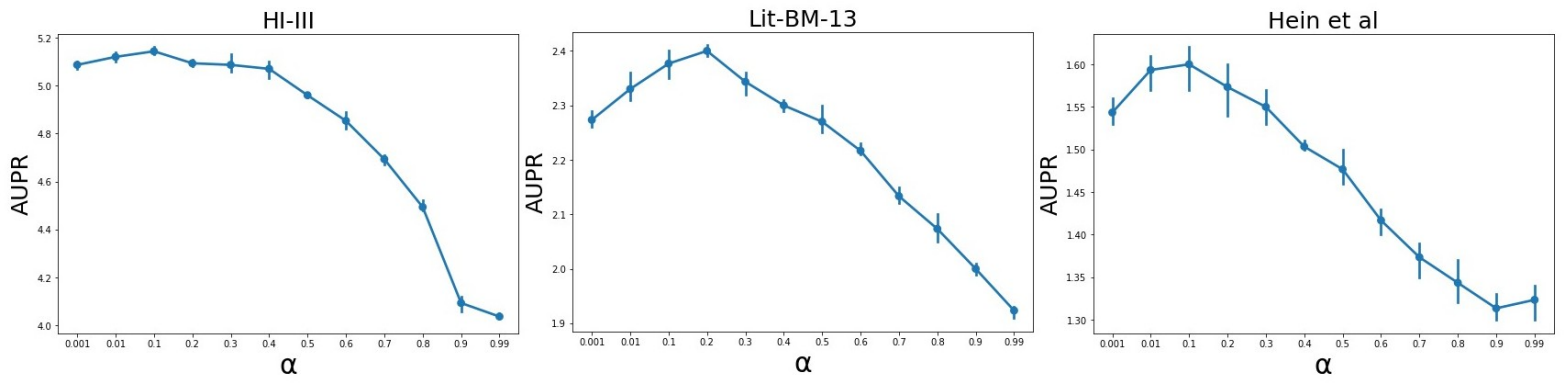
The prediction performance for different values of *α* on three human PPI networks.

**Effect of teleport probability** *α*. Fig 4 shows how the link prediction performance depends on the values of *α* on three human PPI networks. The teleport probability *α* balances the need to preserve locality and larger (higher-order) neighborhood information for each node (protein). The larger the *α*, the more the HO-VGAE model concentrates on the node’s local topological information. Generally, we found a well-balanced point of approximately *α* ∈ [0.1, 0.3] for the range of the neighborhood influencing each node. From Fig 4, we can see that the performance decreases as the value of *α* exceeds 0.4-0.5 because we fail to exploit stronger topological information from higher-order neighborhoods when *α* is too large. In addition, this experiment suggests that the value of *α* should be adjusted for different datasets because different PPI networks require different neighbor ranges. For example, for the HI-III PPI network, when teleport probability *α* = 0.1, the performance is the best. However, the link prediction result of *α* = 0.2 is better than that of *α* = 0.1 on the Lit-BM-13 dataset.

**Fig 5:**
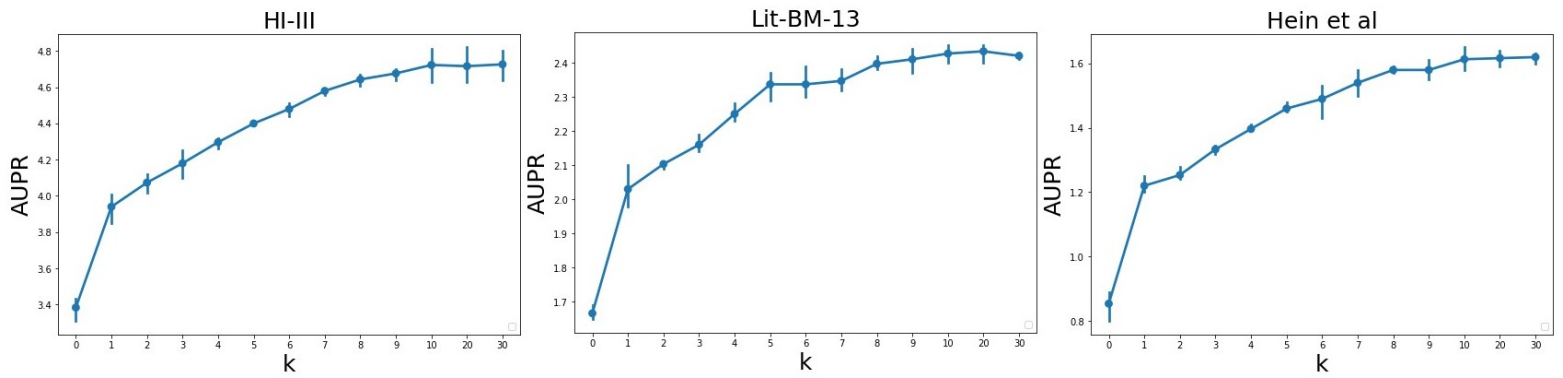
The prediction performance for different power iteration steps *K* on three human PPI networks (*α* = 0.1).

**Effect of the number of power iteration steps** *K*. Fig 5 shows how the number of power iteration steps affects the prediction performance (*α* = 0.1). As clearly shown in the figure, the link prediction performance increases and converges to a stable result as we increase the number of power iteration steps. In detail, when *K* = 0, the node embeddings are totally derived by their own node features *X*. The larger *K* is, the higher-order the neighborhood is that the model explores and propagates graph topological information across. As also shown, when *K* = 10, it is enough to reach the best prediction performance.

### Running time

**Fig 6:**
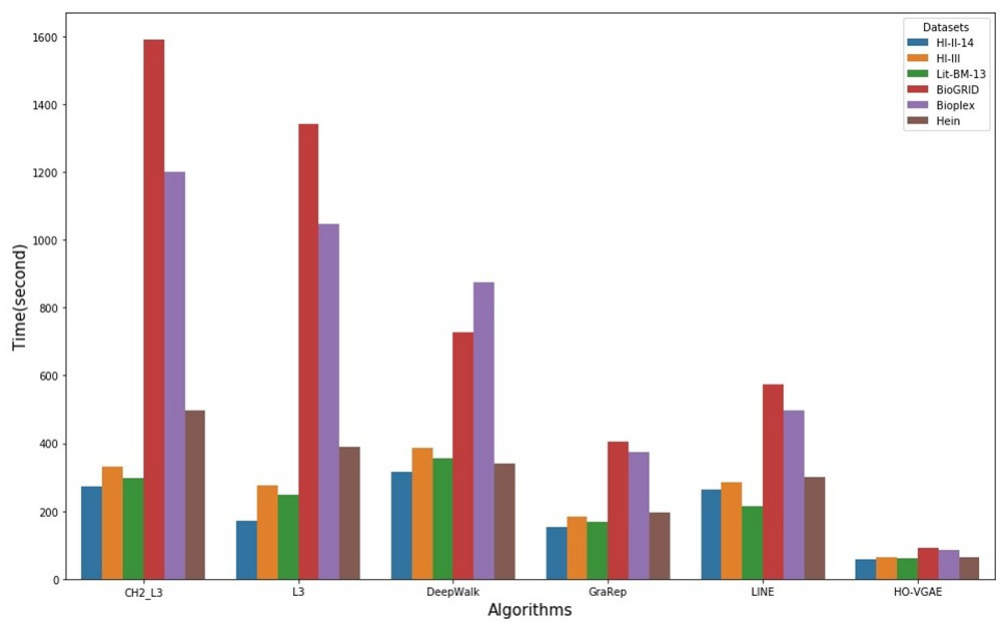
Running times on six datasets for different link prediction alogrithms.

Fig 6 summarized running times on six datasets for different link prediction algorithms. All algorithm codes carried out on a workstation under Windows 10 with 64 GB of RAM and Intel Core i7-8700 processors with 3.20GHz. Note that the running time of all embedding-based methods are computed using the default parameters and do not include the hyper-parameters optimization.

## Conclusion

In this paper, we present a graph embedding-based computational method that can effectively predict missing links in noisy and incomplete PPI networks, with no additional biological information involved. The key idea of our method is that we propose a novel node (protein) embedding model by combining graph convolutional network (GCN) and PageRank because the latter can significantly improve the GCN’s aggregation scheme, which is difficult to extend and explore topological information of networks across higher-order neighborhoods of each node. With our proposed novel higher-order GCN, we further present HO-VGAE as an adaption of VGAE for learning powerful latent embedding from PPI network topology that can be used to discover novel protein interactions. The experimental results demonstrate that our method significantly outperforms all existing graph embedding-based link prediction models in both accuracy and robustness. We believe that the HO-VGAE provides a powerful and necessary tool for automatically expanding the human interactome and for identifying biological legitimate but undetected interactions for experimental determination of PPIs, which is both expensive and time-consuming.

It is worth noting that our model can be easily adapted for many other biological interaction graphs, especially heterogeneous information networks (e.g., drug-protein networks [31] and disease-gene networks [32]), which rely on data obtained from costly high-throughput experimental technologies and are often incomplete and noisy. We leave the adaption of HO-VGAE to further work.

## Supporting information

S1 File Datasets. Six human PPI networks.
(ZIP)

S2 File The lists of top 150 predictions. (ZIP)

S3 File (PDF)

## References

[1] Rual J F, Venkatesan K, Hao T, et al Towards a proteome-scale map of the human protein–protein interaction network. Nature. 2005;437(7062): 1173–1178. 10.1038/nature04209 16189514

[2] Scott D E, Bayly A R, Abell C, et al Small molecules, big targets: drug discovery faces the protein–protein interaction challenge. Nature Reviews Drug Discovery. 2016;15(8): 533 10.1038/nrd.2016.29 27050677

[3] Smits A H, Vermeulen M. Characterizing protein–protein interactions using mass spectrometry: challenges and opportunities. Trends in biotechnology. 2016;34(10): 825–834. 10.1016/j.tibtech.2016.02.014 26996615

[4] Rolland T, Taşan M, Charloteaux B, et al A proteome-scale map of the human interactome network. Cell. 2014;159(5): 1212–1226. 10.1016/j.cell.2014.10.050 25416956 PMC4266588

[5] Hein M Y, Hubner N C, Poser I, et al A human interactome in three quantitative dimensions organized by stoichiometries and abundances. Cell. 2015;163(3): 712–723. 10.1016/j.cell.2015.09.053 26496610

[6] Huttlin E L, Bruckner R J, Paulo J A, et al Architecture of the human interactome defines protein communities and disease networks. Nature. 2017;545(7655): 505–509. 10.1038/nature22366 28514442 PMC5531611

[7] Wodak S J, Vlasblom J, Turinsky A L, et al Protein–protein interaction networks: the puzzling riches. Current opinion in structural biology. 2013;23(6): 941–953. 10.1016/j.sbi.2013.08.002 24007795

[8] Kuchaiev O, Rasajski M, Higham DJ, et al Geometric denoising of protein–protein interaction networks. PLOS Computational Biology. 2009;5:e1000454 10.1371/journal.pcbi.1000454 19662157 PMC2711306

[9] Cannistraci C V, Alanis-Lobato G, Ravasi T. Minimum curvilinearity to enhance topological prediction of protein interactions by network embedding. Bioinformatics. 2013; 29(13): i199–i209. 10.1093/bioinformatics/btt208 23812985 PMC3694668

[10] You Z-H, Lei Y-K, Gui J, et al Using manifold embedding for assessing and predicting protein interactions from high-throughput experimental data. Bioinformatics. 2010;26: 2744–51. 10.1093/bioinformatics/btq510 20817744 PMC3025743

[11] Lei Y-K, You Z-H, Ji Z, et al Assessing and predicting protein interactions by combining manifold embedding with multiple information integration. BMC Bioinformatics. 2012;13(Suppl 7): S3 10.1186/1471-2105-13-S7-S3 22595000 PMC3348017

[12] Lei C, Ruan J. A novel link prediction algorithm for reconstructing protein–protein interaction networks by topological similarity. Bioinformatics. 2012;29(3): 355–364. 10.1093/bioinformatics/bts688 23235927 PMC3562060

[13] Lada A. Adamic and Eytan Adar. Friends and neighbors on the web. Social Networks.2003; 25(3):211–230. 10.1016/S0378-8733(03)00009-1

[14] Wang P, Xu B W, Wu Y R, et al Link prediction in social networks: the state-of-the-art. Science China Information Sciences. 2015;58(1): 1–38. 10.1007/s11432-014-5237-y

[15] Pech R, Hao D, Lee Y L, et al Link prediction via linear optimization. Physica A: Statistical Mechanics and its Applications. 2019;528: 121319 10.1016/j.physa.2019.121319

[16] Lü L., Zhou T. Link prediction in complex networks: A survey. Physica A: statistical mechanics and its applications. 2011;390(6): 1150–1170. 10.1016/j.physa.2010.11.027

[17] Kovács I A, Luck K, Spirohn K, et al Network-based prediction of protein interactions. Nature communications. 2019;10(1): 1–8. 10.1038/s41467-019-09177-y 30886144 PMC6423278

[18] Muscoloni A, Abdelhamid I, Cannistraci C V. Local-community network automata modelling based on length-three-paths for prediction of complex network structures in protein interactomes, food webs and more. BioRxiv [Preprint]. 2018 bioRxiv 346916. https://www.biorxiv.org/content/10.1101/346916.

[19] Bryan Perozzi, Rami Al-Rfou, and Steven Skiena. DeepWalk: online learning of social representations. In: Proceedings of the 20th ACM SIGKDD international conference on Knowledge discovery and data mining. ACM; 2014.p. 701–710.

[20] Aditya Grover and Jure Leskovec. node2vec: Scalable Feature Learning for Networks. In: Proceedings of the 22nd ACM SIGKDD international conference on Knowledge discovery and data mining. ACM; 2016.p. 855–864. 10.1145/2939672.2939754 PMC5108654 27853626

[21] Ou M, Cui P, Pei J, et al. Asymmetric transitivity preserving graph embedding. In: Proceedings of the 22nd ACM SIGKDD international conference on Knowledge discovery and data mining. ACM; 2016.p.1105-1114.

[22] Cao S, Lu W, Xu Q. Grarep: Learning graph representations with global structural information. In: Proceedings of the 24th ACM international on conference on information and knowledge management. ACM; 2015.p.891-900.

[23] Cai H, Zheng V W, Chang K C C. A comprehensive survey of graph embedding: Problems, techniques, and applications. IEEE Transactions on Knowledge and Data Engineering. 2018;30(9): 1616–1637. 10.1109/TKDE.2018.2807452 32614745

[24] Yue X, Wang Z, Huang J, et al. Graph Embedding on Biomedical Networks: Methods, Applications, and Evaluations. arXiv:1906.05017 [Preprint]. 2019. https://arxiv.gg363.site/abs/1906.05017 10.1093/bioinformatics/btz718 PMC7703771 31584634

[25] Su C, Tong J, Zhu Y, et al Network embedding in biomedical data science. Briefings in bioinformatics. 2020;21(1): 182–197. 10.1093/bib/bby117 30535359

[26] Tang J, Qu M, Wang M, Zhang M, Yan J, Mei Q. LINE: Large-scaleInformation Network Embedding. In: Proceedings of the 24th International Conference on World Wide Web. International World Wide Web Conferences Steering Committee; 2015.p.1067–1077.

[27] Wang D, Cui P, Zhu W. Structural deep network embedding. In: Proceedings of the 22nd ACM SIGKDD international conference on Knowledge discovery and data mining. ACM;2016. p.1225-1234.

[28] Kipf T. N., and Welling M. Semisupervised classification with graph convolutional networks. In: ICLR; 2017.

[29] Kipf T N, Welling M. Variational graph auto-encoders. arXiv:1611.07308 [Preprint]. 2016. https://arxiv.gg363.site/abs/1611.07308.

[30] Li Y, Huang C, Ding L, et al Deep learning in bioinformatics: Introduction, application, and perspective in the big data era. Methods. 2019;166: 4–21. 10.1016/j.ymeth.2019.04.008 31022451

[31] Zitnik M, Agrawal M, Leskovec J. Modeling polypharmacy side effects with graph convolutional networks. Bioinformatics. 2018; 34(13): i457–i466. 10.1093/bioinformatics/bty294 29949996 PMC6022705

[32] Singh V, Lio P. Towards Probabilistic Generative Models Harnessing Graph Neural Networks for Disease-Gene Prediction. arXiv:1907.05628 [Preprint]. 2019. https://arxiv.gg363.site/abs/1907.05628

[33] Hamilton W, Ying Z, Leskovec J. Inductive representation learning on large graphs. In: Advances in Neural Information Processing Systems 30. 2017 p.1024–1034.

[34] Veličković P, Cucurull G, Casanova A, et al Graph attention networks. In: ICLR;2018

[35] Schlichtkrull M, Kipf T N, Bloem P, et al. Modeling relational data with graph convolutional networks. In: European Semantic Web Conference. Springer. Cham. 2018: p.593-607.

[36] Li Q, Han Z, Wu X M. Deeper insights into graph convolutional networks for semi-supervised learning. In: Thirty-Second AAAI Conference on Artificial Intelligence; 2018.

[37] Klicpera J, Bojchevski A, Günnemann S. Predict then propagate: Graph neural networks meet personalized pagerank. In: ICLR; 2019.

[38] Lawrence Page, Sergey Brin, Rajeev Motwani, and Terry Winograd. The pagerank citation ranking: Bringing order to the web. Technical report, Stanford InfoLab; 1998.

[39] Kingma D P, Welling M. Auto-encoding variational bayes. arXiv:1312.6114 [Preprint]. 2013. https://arxiv.gg363.site/abs/1312.6114.

[40] Glorot X. and Bengio Y. Understanding the difficulty of training deep feedforward neural networks. In: Proceedings of the thirteenth international conference on artificial intelligence and statistics; 2010: p.249-256.

[41] Luck K, Kim D K, Lambourne L, et al. A reference map of the human protein interactome. BioRxiv [Preprint]. 2019 bioRxiv 605451. 10.1101/605451.

[42] Stark C, Breitkreutz B J, Reguly T, et al BioGRID: a general repository for interaction datasets. Nucleic acids research, 2006, 34(suppl1): D535–D539. 10.1093/nar/gkj109 16381927 PMC1347471

[43] Ahmed A, Shervashidze N, Narayanamurthy S, et al. Distributed large-scale natural graph factorization In: Proceedings of the 22nd international conference on World Wide Web. ACM; 2013: p.37-48.

[44] Belkin M, Niyogi P. Laplacian eigenmaps and spectral techniques for embedding and clustering. In: Advances in neural information processing systems; 2002: p.585–591.

[45] Kingma D P, Ba J. Adam: A method for stochastic optimization. arXiv:1412.6980 [Preprint]. 2014. https://arxiv.gg363.site/abs/1412.6980.

[46] Srivastava N, Hinton G, Krizhevsky A, et al Dropout: a simple way to prevent neural networks from overfitting. The journal of machine learning research. 2014; 15(1): 1929–1958.

