# Supplementary material for: Graph embedding-based novel protein interaction prediction via higher-order graph convolutional network
Source: PLoS One. 2020 Sep 24;15(9):e0238915. doi: 10.1371/journal.pone.0238915 (PMC7514053; doi:10.1371/journal.pone.0238915)
Supplement: S3 File — (PDF) [file pone.0238915.s003.pdf]

Supplementary information: Graph embedding-based novel protein interaction prediction via higher-order graph convolutional network

Ze Xiao[1], Yue Deng[1*]

1 School of Computer Science and Technology, Xidian University, Xi'an, Shaanxi, China

* ydeng@xidian.edu.cn

S1 **Table. Detailed statistics of the datasets.**

Datasets	N	E	D_{av}	ρ
**HI-II-14**	4298	13868	6.212	$1.45E-3$
**HI-III**	5604	23322	8.208	$1.47E-3$
**Lit-BM-13**	5545	11045	3.663	$6.89E-4$
**BioGRID**	12595	66542	10.313	$8.19E-4$
**Bioplex**	10961	56553	10.319	$9.42E-4$
**Hein et al**	5457	28780	10.135	$1.86E-3$

Statistics of datasets, where N is the number of nodes(proteins), E is the number of edges (known interactions), D_{av} is average degree and ρ is the network density.

S2 **Table. Hyper-parameter setting, including candidate hyper-parameter values in grid search and default hyper-parameter values.**

Hyper-parameter values	Candidate	Default
**hidden layer 1 dimensions**	64, 128, 256, 612	256
**hidden layer 2 dimensions**	32, 64, 128, 256	128
**hidden layer 3 dimensions**	16, 32, 64, 128	64
**learning rate**	0.001, 0.01, 0.02, 0.05, 0.1	0.01
**teleport probability** α	0.01, 0.1, 0.2, 0.3, 0.4, 0.5, 0.6, 0.7, 0.8, 0.9	0.1
**power iteration steps** k	1, 2, 3, 4, 5, 6, 7, 8, 9 ,10, 20, 30	10
**co-training edges** t	500, 800, 1000, 1500, 2000, 3000	1500
**dropout rate**	0.1, 0.2, 0.3, 0.5, 0.8	0.5

S3 Table. Overall link prediction performance comparison on six human PPI networks (AUPR). No. positive : No. negative = 1:1.

Method Name	HI-II-14	HI-III	Lit-BM-13	BioGRID	Bioplex	Hein et al	Mean
**HO-VGAE+CH2-L3**	**93.7±0.2**	**95.4±0.2**	**85.5±0.1**	**91.7±0.4**	**84.2±0.8**	**89.4±0.2**	90.0
**HO-VGAE+L3**	**93.7±0.3**	**95.4±0.1**	85.4±0.1	**91.7±0.4**	84.1±0.4	**89.4±0.2**	89.9
**HO-VGAE**	93.5±0.3	**95.4±0.1**	**85.5±0.2**	91.6±0.3	**84.2±0.6**	**89.4±0.2**	89.9
**VGAE+L3**	91.6±0.8	92.0±0.4	81.5±0.4	90.0±0.5	82.3±0.3	88.0±0.2	87.6
VGAE	90.6±0.7	91.7±0.6	79.1±0.4	89.8±0.2	81.8±0.7	87.2±0.5	86.7
GraRep	90.0±0.3	90.3±0.4	77.6±0.4	88.8±0.7	77.0±0.6	87.3±0.3	85.2
PA	85.8±1.0	89.0±1.0	73.5±0.8	87.9±0.1	82.1±0.4	86.2±0.5	84.1
SDNE	88.2±0.7	87.9±0.7	77.1±0.5	87.2±0.7	76.2±0.6	84.8±0.6	83.6
LINE	88.9±1.0	89.3±0.9	76.5±0.4	84.7±0.6	73.6±0.3	83.0±0.6	82.7
L3	83.9±0.6	89.7±0.5	69.5±0.6	83.1±0.8	78.6±0.5	86.9±0.2	81.9
CH2-L3	83.9±0.7	89.9±0.7	68.6±0.3	83.5±0.6	79.0±0.7	86.2±0.6	81.8
HOPE	85.3±0.8	86.0±0.9	73.6±0.9	83.6±1.0	69.1±1.5	83.9±0.7	80.3
DeepWalk	84.5±1.3	84.9±0.7	65.9±0.9	82.1±0.3	73.5±0.7	77.1±0.6	78.0
GF	78.4±0.3	86.7±0.6	70.3±0.4	73.9±0.6	74.4±0.9	75.5±0.5	76.5
node2vec	84.8±1.2	75.9±0.9	63.5±0.8	68.8±0.9	60.8±1.3	64.5±0.6	69.7
CN	65.3±0.9	69.9±0.7	63.1±0.6	66.2±0.5	63.5±0.2	73.9±0.4	67.0
AA	64.5±0.3	70.0±0.5	60.3±0.8	67.1±0.3	65.7±0.9	71.5±0.7	66.5

S4 Table. Overall link prediction performance comparison on six human PPI networks (AUPR). No. positive : No. negative = 1:10.

Method Name	HI-II-14	HI-III	Lit-BM-13	BioGRID	Bioplex	Hein et al	Mean
**HO-VGAE+CH2-L3**	66.9±0.3	73.0±0.6	46.9±0.2	**63.3±0.3**	**51.9±0.5**	52.9±0.6	59.1
**HO-VGAE+L3**	**67.0±0.7**	72.4±0.5	**47.0±0.4**	**63.3±0.4**	**51.9±0.6**	52.7±0.2	59.0
**HO-VGAE**	66.8±0.5	72.3±0.3	46.9±0.3	63.1±0.2	51.8±0.2	52.1±0.1	58.8
CH2-L3	61.9±0.5	**73.4±0.3**	37.9±0.7	59.1±0.3	50.6±0.7	**63.8±0.8**	57.0
L3	62.4±0.5	69.1±0.6	39.4±0.5	56.3±0.6	49.8±0.3	60.6±0.2	56.3
**VGAE+L3**	64.9±0.2	68.2±0.2	36.1±0.7	60.6±0.6	47.6±0.4	50.6±0.7	54.7
VGAE	63.7±0.7	67.7±0.1	35.5±0.2	59.9±0.5	46.5±0.7	49.1±0.2	53.7
GraRep	56.3±0.3	60.5±0.3	34.9±0.4	57.1±1.0	50.2±0.6	41.9±0.2	51.2
LINE	49.3±0.7	51.4±0.7	31.1±0.8	51.2±0.2	48.6±0.4	41.1±0.8	45.4
PA	56.5±0.8	62.4±0.8	30.1±0.4	46.3±0.7	41.2±0.6	46.4±0.4	47.2
HOPE	53.1±0.7	56.4±0.9	31.0±0.6	50.6±0.2	43.3±0.8	37.3±0.9	45.3
SDNE	48.1±0.4	49.6±0.4	30.1±0.4	47.2±0.1	46.3±1.0	39.6±0.3	43.5
GF	45.2±0.5	50.4±0.5	24.1±0.3	39.2±0.7	37.9±0.5	26.0±0.4	41.3
DeepWalk	37.9±0.7	49.3±0.4	17.0±0.4	37.5±0.8	36.2±0.2	33.6±0.5	35.3
CN	29.5±0.8	35.4±0.9	27.8±0.5	29.7±0.4	28.3±0.8	42.8±0.5	32.3
AA	28.8±0.2	37.3±0.5	23.5±0.5	30.8±0.9	30.5±0.2	41.7±0.6	32.1
node2vec	30.7±2.0	42.1±0.8	13.5±0.6	33.2±0.8	33.2±0.9	30.1±0.5	30.5

S5 Table. Hadamard product based and concatenation based graph embedding performance (AUPR). All unknown pairs were treated as negative examples.

Method Name	category	HI-II-14	HI-III	Lit-BM-13	BioGRID	Bioplex	Hein et al	Mean
GraRep	Hadamard product	2.0±0.3	2.4±0.0	1.0±0.0	1.5±0.1	1.4±0.1	0.9±0.1	1.5
GraRep	Concatenation	2.0±0.2	2.3±0.1	1.0±0.1	1.6±0.1	1.6±0.0	0.9±0.1	1.6
SDNE	Hadamard product	1.0±0.1	1.0±0.0	0.5±0.0	1.2±0.1	1.0±0.0	1.2±0.0	0.98
SDNE	Concatenation	1.1±0.2	1.0±0.1	0.5±0.0	1.3±0.0	1.0±0.0	1.2±0.0	1.0
LINE	Hadamard product	1.2±0.0	1.1±0.0	0.6±0.0	1.1±0.1	1.2±0.0	0.7±0.0	0.98
LINE	Concatenation	1.2±0.0	1.1±0.0	0.6±0.0	1.1±0.0	1.2±0.0	0.7±0.0	0.98
GF	Hadamard product	1.3±0.1	1.7±0.3	0.4±0.0	0.9±0.1	0.5±0.0	0.5±0.0	0.88
GF	Concatenation	1.4±0.2	1.7±0.2	0.4±0.0	0.6±0.0	0.9±0.0	0.5±0.0	0.92
DeepWalk	Hadamard product	1.0±0.0	1.8±0.1	0.2±0.0	1.2±0.3	0.8±0.0	0.6±0.0	0.8
DeepWalk	Concatenation	1.0±0.1	1.8±0.1	0.2±0	1.0±0.1	0.8±0.1	0.8±0.0	0.93
node2vec	Hadamard product	0.9±0.0	1.5±0.2	0.2±0.0	1.0±0.1	0.8±0.1	0.6±0.0	0.87
node2vec	Concatenation	0.8±0.0	1.5±0.1	0.2±0.0	1.0±0.0	0.7±0.0	0.8±0.0	0.80

S6 Table. Hadamard product based graph embedding performance (Precision@k).

Method Name	category	HI-II-14	HI-III	Lit-BM-13	BioGRID	Bioplex	Hein et al	Mean
GraRep	Hadamard product	12.0±0.3	13.6±0.6	7.9±0.3	9.9±0.1	9.2±0.1	10.6±0.2	10.53
GraRep	Concatenation	12.6±0.1	13.6±0.6	7.9±0.3	9.8±0.3	9.2±0.1	10.6±0.2	10.86
LINE	Hadamard product	10.6±0.4	10.5±0.3	6.2±0.2	8.1±0.1	8.2±0.1	9.2±0.1	8.80
LINE	Concatenation	11.4±0.2	11.0±0.1	6.1±0.3	8.5±0.3	8.6±0.3	9.1±0.3	9.11
SDNE	Hadamard product	8.0±0.1	10.1±0.4	3.9±0.1	7.0±0.2	7.2±0.3	7.3±0.0	7.25
SDNE	Concatenation	8.5±0.3	10.2±0.2	4.1±0.2	7.1±0.1	7.4±0.1	7.6±0.2	7.48
GF	Hadamard product	7.3±0.4	6.8±0.3	7.7±0.2	4.7±0.2	4.4±0.1	7.0±0.4	6.33
GF	Concatenation	7.7±0.2	6.9±0.2	7.7±0.5	5.1±0.3	4.4±0.1	7.3±0.1	6.51
DeepWalk	Hadamard product	6.5±0.0	8.3±0.1	3.4±0.0	4.7±0.0	6.1±0.1	5.6±0.0	5.76
DeepWalk	Concatenation	6.7±0.3	8.9±0.1	3.3±0.1	5.0±0.2	6.0±0.1	5.8±0.2	5.95
node2vec	Hadamard product	4.9±0.2	8.1±0.3	1.6±0.1	3.0±0.2	5.3±0.4	4.3±0.3	4.45
node2vec	Concatenation	4.8±0.4	8.5±0.3	1.5±0.0	3.1±0.2	5.3±0.4	4.3±0.3	4.58

S7 Table. Link performance with and without using DeepWalk to generate node features (AUPR). All unknown pairs were treated as negative examples.

Method Name	category	HI-II-14	HI-III	Lit-BM-13	BioGRID	Bioplex	Hein et al	Mean
HO-VGAE	With DeepWalk	4.5±0.1	5.6±0.4	2.4±0.1	5.5±0.1	4.6±0.0	1.6±0.17	4.03
HO-VGAE	Without DeepWalk	4.7±0.3	5.1±0.2	2.4±0.0	5.7±0.2	4.7±0.1	1.6±0.0	4.10
VGAE	With DeepWalk	3.5±0.1	4.7±0.3	1.4±0.0	4.7±0.1	3.6±0.0	1.3±0.1	3.18
VGAE	Without DeepWalk	3.4±0.1	4.4±0.1	1.4±0.1	4.6±0.2	3.7±0.1	1.3±0.1	3.13

S8 Table. Link performance with and without using DeepWalk to generate node features (Precision@k).

Method Name	category	HI-II-14	HI-III	Lit-BM-13	BioGRID	Bioplex	Hein et al	Mean
HO-VGAE	With DeepWalk	17.0±0.3	18.5±0.3	10.9±0.2	10.3±0.1	12.5±0.6	12.1±0.5	13.55
HO-VGAE	Without DeepWalk	17.7±0.1	18.5±0.4	10.6±0.5	10.4±0.3	12.4±0.3	12.8±0.3	13.70
VGAE	With DeepWalk	15.5±0.6	16.0±0.1	8.9±0.5	9.1±0.2	10.3±0.2	10.7±0.6	11.75
VGAE	Without DeepWalk	15.2±0.4	16.6±0.3	8.9±0.2	9.0±0.2	10.1±0.3	11.2±0.4	11.83
